# Chestnuts in Fermented Rice Beverages Increase Metabolite Diversity and Antioxidant Activity While Reducing Cellular Oxidative Damage

**DOI:** 10.3390/foods12010164

**Published:** 2022-12-28

**Authors:** Jing Zou, Yinghong Hu, Kuo Li, Yang Liu, Miao Li, Xinyuan Pan, Xuedong Chang

**Affiliations:** 1 Department of Food Science and Technology, Hebei Normal University of Science and Technology, Qinhuangdao 066600, China; 2 Engineering Research Center of Chestnut Industry Technology, Ministry of Education, Qinhuangdao 066000, China; 3 Chestnut Industrial Technology Research Institute of Hebei Province, Chengde 067699, China

**Keywords:** chestnut, lactic acid bacteria beverage, technology, antioxidant activity, Caco-2 cell

## Abstract

Foods containing chestnuts (*Castanea mollissima* Blume) are relatively uncommon, despite the high nutrient and starch contents and purported health benefits. In this study, we examine the flavor-related metabolites, volatile compounds, and amino acids in a traditional glutinous rice fermented beverage supplemented with chestnuts as a fermentation substrate for lactic acid bacteria (LAB). Changes in antioxidant activity towards free radicals and effects on cellular oxidative stress are compared between beverages with or without chestnuts. The fermented chestnut-rice beverage (FCRB) has higher sensory scores and a wider range of volatiles and flavor-related compounds (74 vs. 38 species compounds), but lower amino acid contents, than the traditional fermented glutinous rice beverage (TFRB). In free radical scavenging assays, the FCRB exhibits higher activity than the TFRB in vitro. Furthermore, while neither beverage induces cytotoxity in Caco-2 cells at concentrations up to 2 mg/mL, pretreatment with the FCRB results in lower rates of apoptosis and necrosis and higher overall viability in cells with H_2_O_2_-induced oxidative stress compared to pretreatment with the TFRB. The enhanced reactive oxygen species neutralization in vitro and protection against oxidative damage in cells, coupled with increased diversity of volatiles and flavor-related metabolites of LAB, support the addition of chestnuts to enhance flavor profile and antioxidant properties of fermented functional foods.

## 1. Introduction

Market research has shown that many consumers do not purchase foods based solely on nutritional needs, but rather increasingly seek foods purported to provide functional effects, such as improving immune response, regulating physiological processes or circadian rhythms, preventing diseases, or promoting their recovery. This trend has thus dramatically increased the market demand for functional foods [1]. Among functional foods, the number and types of functional beverages, especially non-dairy beverages, have relatively exploded due in part to current trends in lifestyle choice (e.g., vegetarianism and veganism) [2], avoidance of allergic reactions or food intolerances (e.g., lactose intolerance [3], gluten intolerance [4]), and diet-associated diseases (such as cardiovascular disease or high blood pressure [5,6]). Based on this growing demand, considerable research attention is now focused on identifying and developing new functional foods, dietary supplements, and food-based pharmaceutical formulations.

This new generation of functional foods includes chestnuts, which have a high nutrient content accompanied by a unique flavor, but remains under-utilized as a component in functional foods [7,8]. Chestnuts have considerable potential for the improvement of established food products or for the development of new products due to its high contents of complex carbohydrates, proteins, soluble fiber, vitamins, and minerals, in addition to a relatively wide range of antioxidant and fatty acid metabolites [7,9]. Moreover, the high starch content makes chestnuts amenable to processing through fermentation, which could further enhance the content and diversity of its bioactive components, and consequently, its potential probiotic functions [10,11].

Among the possible approaches to chestnut processing, lactic acid bacteria (LAB) fermentation represents an effective approach for developing chestnut-based probiotic foods, since LAB have been shown to improve the bioavailability and bioactivity of plant metabolites [12,13], while adding other, potentially beneficial, bacterial metabolites. In addition, foods produced by LAB fermentation have been reported to confer a wide range of probiotic functions, such as regulating the immune system [14], reducing cholesterol levels [15], promoting the growth of beneficial intestinal flora while inhibiting intestinal colonization by pathogenic bacteria [16], alleviating constipation [17], conferring anti-aging and antioxidant properties, and reducing the risk of tumors [18]. Previous studies have shown that chestnuts can serve as a suitable substrate for the growth of lactic acid bacteria, which may in turn enhance their sensory properties [19], either through the accumulation of LAB and their metabolic products, or by altering the chemical composition during fermentation [20].

Therefore, we hypothesized that LAB could be used to ferment chestnut-based functional foods. In this study, chestnuts were added as a substrate to semi-solid-state LAB fermentation of the traditional fermented rice beverage (TFRB) to produce a fermented chestnut-glutinous rice beverage (FCRB). The flavor characteristics, nutrient contents, including total sugar, amino acids, and organic acids, were quantified, and the flavor-related compounds of FCRB and TFRB were investigated. Additionally, the protective effects of fermented chestnut-rice beverage and TFRB against hydrogen peroxide (H_2_O_2_)-induced oxidative damage in Caco-2 cells were also quantified. This work provides a valuable resource for understanding the effects of adding chestnuts as a raw fermentation substrate to traditional foods, and illustrates how flavor profile can be enhanced in foods produced using LAB fermentation.

## 2. Materials and Methods

### 2.1. LAB Strains, Substrates, and Fermentation Conditions

The glutinous rice was obtained from Jilin Longyuan Rice Industry Co., Ltd. (Jilin, China). The Chinese chestnuts (*Castanea mollissima* Blume) were obtained from a farm in QianXi county, Hebei province, China. The *Rhizopus oryzae* sweet rice leavening agent was purchased from Angle Yeast Co., Ltd. (Yichang, China), and the *Pediococcus pentosaceus* strains DH16, DH20 and DH24 were isolated from jiuqu starters obtained from DaZhu Jiuqu company (Dazhou, China) [21]. The three LAB strains were cultivated in MRS broth (Land Bridge Technology Co. Ltd., Beijing, China) at 37 °C for 12 h, then adjusted to a cell density of 1 × 10^8^ CFU/mL and mixed in a 1:1:1 (*v*/*v*) ratio before inoculation into fermentation.

The glutinous rice was washed and soaked for 12 h at 20 °C, after which the water was drained. The soaked glutinous rice was steamed for 30 min, then cooled to 30 °C, at which point 0.4% (*w*/*w*) leavening agent and 3.0% (*v*/*w*) lactic acid bacteria culture were added. Following 24 h of fermentation at 30 °C, sterile water was added in a 1:4 rice: water (*w*/*w*) ratio. The mixture was homogenized by a pulp refiner, and the homogenous mixture, i.e., the final TFRB product, was bottled and sterilized at 85 °C for 20 min.

The FCRB fermentation process was similar to that for the TFRB, except that shelled chestnut kernels were chopped to approximately the size of rice grains and added to the glutinous rice at a 1:4 (*w*/*w*, dry weight) ratio before steaming. This fermentation process was used to produce three separate batches of the FCRB and TFRB each.

### 2.2. Determination of Organic Acids

To detect organic acids, 1 mL samples of each fermented beverage were mixed with 9 mL distilled water, then centrifuged for 10 min at 7100× *g*. The supernatants were collected and stored at 4 °C before analysis. An Agilent 1260 Series HPLC system (Agilent Technologies, Foster City, CA, USA) equipped with a pump and a UV/VIS detector (SPD-20A) set to monitor 210 nm was used for organic acid detection. Separation was carried out on an Aminex HPX-87H column (300 × 7.8 mm) (Bio-Rad) at 55 °C. The analytical conditions were as follows: flow 0.3 mL min^−1^, eluent 0.045 M H_2_SO_4_ with 6% acetonitrile (*v*/*v*).

### 2.3. Determination of Total Phenols and Total Flavonoids

To determine total phenol contents, the methods of Lingua et al. [22] were followed with some modifications. Briefly, each reaction included 250 µL samples of fermented beverage, 12.5 mL distilled water, 1.25 mL Folin–Ciocalteu reagent, and 5 mL Na_2_CO_3_ (20%, *w*/*v*). The other operations were performed according to the methods described by Linggua et al.[22].

For total flavonoids, 1 mL samples of each beverage were mixed with 1 mL NaNO_3_ (5%, *w*/*v*) and incubated for 6 min. Then the other analyzing operation to determine the total flavonoid contents was performed according to the method described by Lingua et al. [22], with some modifications.

### 2.4. Analysis of Free Amino Acid (FAAs)

The FAA contents in each sample were determined according to the protocols of Chen et al. [23], with some modifications. Briefly, FAAs were analyzed with an automatic amino acid analyzer (S-4330D, Sykam, Germany) using an equivalent volume of ethanol to precipitate proteins and/or peptides for 2 h at 4 °C. Samples were then centrifuged at 8000× *g* (15 min, 4 °C), and filtered through a 0.22 μm filter membrane. The filtrates were collected and stored at 4 °C for further analysis.

### 2.5. HS-SPME-GC/MS-O Determination of the Volatile Profile

Volatile organic compound (VOC) profiles were determined following procedures described by Zou et al. [11], with some modifications. Samples of the FCRB and TFRB were stored at 4 °C before analysis, and 1 µL 2-octanol internal standard and 2 g NaCl were added to each 8 mL sample in a 20 mL vial and tightly capped with a PTFE/silicone septum. Carboxen/polydimethylsiloxane (CAR/PDMS) SPME fibers (75 µm, Fused Silica 24 Ga, Manual Holder, 3 pk, Supelco, Bellefonte, PA, USA) were used for VOC extraction at 60 °C for 50 min. Compounds were desorbed for 5 min at 280 °C in splitless mode, using a 0.75 mm dedicated SPME liner.

Gas chromatography conditions, MS operating conditions, and qualitative and semi-quantitative analysis were all performed according to the method described by Zou et al. [11].

### 2.6. Determination of the Antioxidant Ability

For antioxidant assays, samples of each fermented beverage were dried via vacuum freeze-drying, and different quantities of each fermented beverage were diluted in distilled water to a final concentration of 1 g/L. Different volume samples of each fermented beverage were used to perform antioxidant or free radical scavenging assays.

#### 2.6.1. Determination of the DPPH^•^ Radical Scavenging Activities

For both beverages, DPPH^•^ scavenging activity was measured using the same method as that reported by Zou et al. [11], with minor modifications. Briefly, 0.01% DPPH^•^ was dissolved in ethanol (*w*/*v*), and 2 mL of this solution was mixed with different volumes of the fermented beverage to a final weight of 100, 200, 300, 400, or 500 µg, and each sample was brought to a final volume of 4 mL with distilled water. Then, the specific operation process was performed according to the method given by Zou et al. [11]. All assays were performed in triplicate. The scavenging activity was calculated as follows:

(1) $$\mathrm{Scavenging}\;\mathrm{activity}\;(\%)=\;\left(1-\frac{A_{sample+DPPH}-A_{sample}}{A_{DPPH}}\right)\times 100\%$$

where *A_sample+DPPH_* represents DPPH^•^ with beverage samples, *A_sample_* represents beverage samples with ethanol instead of DPPH^•^, and *A_DPPH_* represents DPPH^•^ solution without beverage samples.

#### 2.6.2. Ferric Reducing Antioxidant Power (FRAP)

FRAP assays were performed according to procedures described by Barros et al.[24], with some modifications. First, the FRAP reagent was prepared according the method given by Barros et al.[24]. For each reaction, 100 μL aliquots of the fermented beverage samples were mixed with 2.9 mL of the FRAP reagent, and incubated at 37 °C for 30 min. The change in absorbance at 593 nm was recorded against acetate buffer (pH 3.6), using a UV-VIS Spectrophotometer 1204 (Shimadzu, Kyoto, Japan). Aqueous solutions of FeSO_4_•7H_2_O (100 to 1000 μM) were used to calibrate the instrument, and results were expressed as the FRAP values (μM Fe (II)) of each sample. All determinations were conducted in triplicate.

#### 2.6.3. Hydroxyl Radical (•OH) Scavenging Activity

To determine the hydroxyl radical scavenging activity of each fermented beverage sample, the protocols given by Barros et al. [24] were used with some modifications. Each sample contained different amounts of the fermented beverage, ranging from 0.1–0.5 mL in distilled water to a final volume of 1 mL, each of which was then added to a reaction mixture (the specific composition has been described in the method section of the paper published by Barros et al. [24]). The samples were mixed and reacted at 37 °C for 60 min. Absorbance was measured at 536 nm using ascorbic acid (1 g/L) as a positive control.

Each sample was evaluated three times, and the hydroxyl radical scavenging activity was calculated as follows:

(2) $$\mathrm{Hydroxyl}\;\mathrm{radical}\;\mathrm{scavenging}\;\mathrm{co-efficient}\;(\%)=\frac{A_{sample}-A_{damage}}{A_{blank}-A_{damage}}\times 100\%$$

where *A_blank_* represents distilled water without the fermented beverage sample or H_2_O_2_, *A_damage_* represents H_2_O_2_ without the fermented beverage sample, and *A_sample_* includes the fermented beverage with H_2_O_2_.

#### 2.6.4. ABTS^•+^ Radical Scavenging Assay

The capacity for scavenging ABTS^•+^ radicals of each fermented beverage was measured according to the methods published by Ye [25], with some modifications. For these assays, a working solution was prepared by mixing equal volumes of 7.4 mM ABTS^•+^ and 2.6 mM potassium persulfate stock solution, followed by 12 h incubation in the dark at room temperature. Distilled water was added to different volumes of the fermented beverage samples, from 10–90 µL, to reach a sample volume of 0.1 mL. These diluted samples were then mixed with 4.9 mL of the ABTS^•+^ working solution via vortexing. After 5 min incubation at room temperature under dark conditions, the absorbance at 734 nm was recorded. Distilled water served as the blank or negative control, while 1 g/L ascorbic acid in solution served as the positive control. Three separate reactions were conducted for each sample, and the ABTS^•+^ radical scavenging activity was calculated according to the formula below:

(3) $$\mathrm{Scavenging}\;\mathrm{rate}\;(\%)=\left(1-\frac{A_{sample}}{A_{blank}}\right)\times 100\%$$

### 2.7. Determination of Cellular Antioxidant Activity (CAA)

#### 2.7.1. Cytotoxicity Assays

To determine their potential cytotoxicity, the samples of each fermented beverage were dried via vacuum freeze-drying, and different quantities of each fermented beverage were diluted in the complete EMEM medium (30-2003, ATCC, Rockville, MD, USA) to final concentrations of 0.05 mg/mL, 0.1 mg/mL, 0.25 mg/mL, 0.5 mg/mL, 0.75 mg/mL, and 2 mg/mL. Cytotoxicity towards Caco-2 cells was determined by adding each of the beverages to culture the medium followed by MTT (3-(4,5)-dimethylthiahiazo (-z-y1)-3,5-di- phenytetrazoliumromide) assays. First, Caco-2 human colon cancer cells (ATCC, Rockville, MD, USA), which are physiologically similar to small intestinal epithelial cells, were cultured in complete EMEM medium containing penicillin (final concentration of 100 U/mL), streptomycin (Sigma-Aldrich, St. Louis, MO, USA; final concentration of 100 μg/mL), and 10% FBS (Fetal Bovine Serum, Hyclone Logan, UT, USA). Caco-2 cells were harvested during the logarithmic growth stage and adjusted to a cell density of 5 × 10^3^ cells per well in 180 µL, inoculated to each well of a 96-well plate. Plates were incubated at 37 °C under 5% CO_2_ modified atmosphere until the cells were 90% fused, after which the cells were synchronized by 2 h incubation with serum-free EMEM medium.

The synchronized cells were then treated for 24 h with different concentrations of each beverage, as prepared above. After treatment, the supernatants were carefully removed and 90 μL of EMEM medium and 10 μL of MTT solution (Solarbio, Beijing, China) were added. Cells were then incubated for 4 h under the same growth conditions as above. Supernatants were again removed and discarded, and 110 µL formazan dissolving solution was added to each well, followed by 10 min incubation on a plate shaker by slowly increasing speed to a maximum of 900 shakes/min to dissolve the crystals. The absorbance at 490 nm was read using a multi-well scanning spectrophotometer (ELIASA LB941, Berthold Technologies, Stuttgart, Germany) and the absorbance was compared with that of the control group to determine whether there are effects of each beverage on the cellular oxidoreductase activity as a measure of viability.

#### 2.7.2. H_2_O_2_-Induced Oxidative Stress Assays

To determine the effects of each beverage on cellular response to oxidative stress, Caco-2 cells collected in the logarithmic growth phase were adjusted to a cell density of 5 × 10^3^ cells per well in 200 µL and synchronized in serum-free EMEM medium for 2 h. The synchronized Caco-2 cells were then separated into untreated control, H_2_O_2_-stressed control, and test groups. Synchronized cells in the test groups were incubated in serum-free EMEM medium, and freeze dried powders of the fermented beverages were added to reach final concentrations of 0.25 mg/mL (low-dose group), 1 mg/mL (middle-dose group), or 2 mg/mL (high-dose group), and then incubated at 37 °C for 24 h. Alternatively, synchronized Caco-2 cells in the untreated controls were cultured in serum-free EMEM medium at 37 °C for 26 h with no added H_2_O_2_ and/or fermented beverages, while synchronized Caco-2 cells in the stressed controls were incubated for 24 h in serum-free EMEM medium at 37 °C, then incubated for another 2 h in new EMEM medium containing 1 mmol/L H_2_O_2_. After treatment, supernatants were carefully removed and 90 μL of EMEM medium was added with 10 μL of MTT solution (Solarbio, Beijing, China). Plates were then incubated for 4 h at 37 °C with 5% CO_2_ modified atmosphere, after which supernatants were discarded and replaced with 110 µL formazan dissolving solution. Plates were again incubated with shaking for 10 min at 100 shakes/min to dissolve all crystals, and NADH reducing activity was quantified by using a plate reader, as described in Section 2.7.1.

#### 2.7.3. Intracellular Reactive Oxygen Species (ROS) Accumulation Assays

To measure the effects of each beverage on cellular ROS neutralization under H_2_O_2_-induced oxidative stress, synchronized Caco-2 cells were treated according to the same process as described in Section 2.7.2. After 2 h treatment with 1 mM H_2_O_2_ at 37 °C, supernatants were discarded and 2 mL 0.25% (*w*/*v*) EDTA-free trypsin solution was added for 30 s. Caco-2 cells were then collected via centrifugation at 250× *g* for 5 min at 4 °C. Then, 1.5 mL of 5 μM DCFH-DA working solution was added to each sample and incubated in the dark for 20 min at 37 °C. After incubation, cells were centrifuged at 250× *g* for 10 min, and the staining solution was discarded. Cell pellets were washed twice with 200 μL PBS, and then finally resuspended in 200 μL PBS to detect fluorescence intensity on enzyme-linked immunometric meter (ELISA LB941, Berthold Technologies, Stuttgart, Germany) at 488 nm excitation/525 nm emission.

#### 2.7.4. Intracellular Antioxidant Enzyme Activity and Glutathione (GSH) Content Determination

After preparing cells according to the same process as described in Section 2.7.2 and separation into the treatment and control groups, stress was induced by 2 h exposure to 1 mmol/L H_2_O_2_. Cells were then digested with 2 mL 0.25% (*w*/*v*) trypsin solution for 30 s and collected via 5 min centrifugation at 250× *g* at 4 °C. Cell density was adjusted to 1 × 10^6^ cfu/mL, then incubated with reaction solution following instructions accompanying the total antioxidant capacity microplate assay kit (Nanjing Jiancheng Biological Engineering Company, Nanjing, China). The absorbance at 405 nm was then measured using a plate reader with a diameter of 0.5 cm. In addition, a bicinchoninic acid protein assay kit (Nanjing Jiancheng Biological Engineering Company, Nanjing, China) was used to determine the total protein content and to calculate the total antioxidant capacity of the two fermented beverages according to the instruction of the total antioxidant capacity microplate assay kit.

A total superoxide dismutase (T-SOD) assay kit (WST-1 method) (Nanjing Jiancheng Biological Engineering Company, Nanjing, China) was used to determine the total superoxide dismutase activity after H_2_O_2_ treatment and adjusting Caco-2 cell density to 1 × 10^6^ cfu/mL following the manufacturer’s instructions. A unit of SOD activity (U/mg protein) was defined as the amount enzyme corresponding to a 50% SOD inhibition rate. A catalase (CAT) assay kit (visible light; Nanjing Jiancheng Biological Engineering Company, Nanjing, China) was used to measure catalase activity in the different treatment groups according instructions accompanying the kit. A unit of catalase activity (U/mg protein) was defined as the amount of catalase in 1 mg of cells required to degrade 1 µmol of H_2_O_2_ in 1 s. In addition, glutathione (GSH) contents were measured using a reduced glutathione (GSH) assay kit (Nanjing Jiancheng Biological Engineering Company, Nanjing, China) following the kit instructions.

#### 2.7.5. Cell Apoptosis

To measure apoptosis in oxidatively stressed cells treated with the fermented beverages, Caco-2 cells were grown in 96-well plates with EMEM medium and different amounts of either fermented beverage (0.25 mg/mL, 1 mg/mL, 2 mg/mL) at 37 °C for 24 h. Cells were treated or not with H_2_O_2_ (1 mM) for 2 h at 37 °C, then washed with pre-cooled PBS at 4 °C, and centrifuged at 110× *g* for 5 min at 4 °C. Supernatants were discarded and 5 μL of Annexin V (Invitrogen, Carlsbad, CA, USA) and 1 μL of 100 μg/mL PI (Invitrogen, Carlsbad, CA, USA) working stocks were added to each cell suspension, then incubated for 15 min at room temperature. Apoptosis was determined using DxFLEX flow cytometry (Beckman-Coulter, Bria, CA, USA).

### 2.8. Statistical Analysis

Each experiment was repeated three times. The data were expressed as the mean plus or minus standard deviation (X ± SD). Statistically significant differences between groups were determined with the one-tail T test using SPSS version 20.0 software, with *p* ≤ 0.05 represented by “#”, “*” or different letters in figures, and *p* ≤ 0.01 indicated by “##” or “**” markers.

## 3. Results and Discussion

### 3.1. Physicochemical Characteristics

#### 3.1.1. Organic Acids

Organic acids produced during the fermentation, which can lower the beverage pH and extend its shelf life, may also confer several purported health benefits such as detoxification and promoting healthy bowel function [26,27]. HPLC-based quantification of various organic acid species and total contents in the FCRB and TFRB identified three main organic acid species in the FCRB. In particular, lactic acid was the most abundant organic acid in both the FCRB (1573.11 ± 91.36 mg/L) and the TFRB (1487.33 ± 101.15 mg/L), while citric acid was the second most abundant acid in the FCRB (230.28 ± 19.85 mg/L), at significantly higher levels, than that observed in the TFRB (109.35 ± 9.92 mg/L). Fumaric acid was the least abundant of the identified organic acids, detected at 1.61 ± 0.25 mg/L in the FCRB and 2.35 ± 0.21 mg/L in the TFRB. Organic acids can act as antimicrobial agents, in some cases, by interfering with the maintenance of cell membrane potential, inhibiting active transport, reducing intracellular pH, or by inhibiting a variety of metabolic functions, which together limit the growth of many pathogens and spoilage bacteria [28], including both Gram-positive and Gram-negative bacteria as well as yeast and molds [29]. Since the processing, fermentation conditions, and starter cultures were all the same, and the citric acid content increased in cooked chestnuts [30], we hypothesized that the significant difference in the citric acid content between the beverages was likely due to the addition of chestnuts.

#### 3.1.2. Total Phenol and Flavonoid Profiles

Previous studies have shown that chestnuts are rich in polyphenols [31]. To compare the phenolic profiles between the FCRB and TFRB, the total phenolic contents of each fermented beverage were determined by using the Folin–Ciocalteu method, with gallic acid serving as an internal standard for phenol quantitation (Table S1). The results showed that the total polyphenol contents were significantly greater in the FCRB (131.17 ± 8.25 µg/mL) than in the TFRB (90.14 ± 5.43 µg/mL). Next, quantification of the total flavonoid contents through a chromogenic method, using rutin as a standard (Table S1), indicated that 43.14 ± 1.26 µg/mL of flavonoids were present in the FCRB, which was significantly higher than that in the TFRB (30.24 ± 2.45 µg/mL) (*p* < 0.05). Together with the results of phenolic quantification, these data showed that adding a proportion of chestnuts to the fermentation process increased the contents of metabolites in the final product. The increased phenolic contents in the FCRB might result in the release of bound phenolics, based on previous studies that showed bound polyphenols can form glycosidic of ester bonds with dietary fiber [32], and that these bonds may be hydrolyzed during microbial fermentation, releasing the bound polyphenolics [32,33], an effect previously reported in fermented chestnut-rice wine [11].

#### 3.1.3. Free Amino Acid (FAA) Contents in the FCRB and TFRB

Amino acids are not only important from a nutritional perspective, but also play a crucial role as precursors of aroma compounds that can directly contribute to the flavor of fermented foods [34]. An amino acid analyzer was used to determine the amino acid species and contents in each fermented beverage. This analysis revealed that the FCRB contains the same suite of 17 peptide amino acids, but at a significantly lower total amino acid concentration in the FCRB than that in the TFRB (2215.775 vs. 3786.911 mg/L; *p* < 0.05) (Table 1). Only Asp was significantly higher and Glu, Cys, and His were not significantly lower in the FCRB, while the concentrations of the other 13 amino acids were all significantly lower in the FCRB than in the TFRB. In addition, the total contents of the eight essential amino acids (EAAs) in the FCRB was approximately 55.80% that in the TFRB, although the ratio of total umami and sweet FAAs to bitter FAAs in the FCRB was 1.183, which was 26.66% higher than that in the TFRB. The amino acid composition of the fermented foods depends on the starting materials and processing methods. The protein content in chestnuts is lower than that in rice, thus resulting in a lower total free amino acid content in the FCRB. However, the proportion of flavor-related amino acids in the FCRB is different from that in the TFRB, which might change the flavor of FCRB, as the amino acids are an important contribution to the flavor of fermented beverages [35].

### 3.2. Quantification of Volatile Compounds

In order to identify the flavor-related compounds in the FCRB and TFRB, volatile compounds in both the beverages were quantified (Table 2). A total of 74 compounds were detected, including 18 olefins, 11 esters, 22 benzenes, eight alcohols, five aldehydes, three ketones, three alkanes, one organic acid, and three other compounds. By contrast, only 38 volatile compounds were detected in the TFRB, including 10 alcohols, nine aldehydes, five olefins, five alkanes, three ketones, three esters, one benzene, one organic acid, and one phthalan. In addition to differences in the number of detectable species, the concentrations and specific volatile metabolites in the TFRB were also markedly different from that in the FCRB. In the FCRB, alcohols comprised the largest proportion (30.72%) of total flavor-related compounds. Among the eight most abundant alcohol species in the FCRB, 2-octanol, which confers a pungent fruity aroma, was the most abundant, followed by benzene ethanol, which reportedly presents the aroma of rose [36], and 2-nonanol, which is a main flavor compound in uncooked corn [37]. Notably, only three ester species were detected in the TFRB, but together accounted for the largest proportion of potential flavor-related volatiles (41.59%). The most abundant of these was ethyl hexadecanoate, a high molecular weight fatty acid ester with a waxy odor that has been identified as a crucial volatile flavor component of Luzhou liquor [38]. The other two high molecular weight esters included ethyl tetradecanoate and methyl linolate, which were likely produced through fungal metabolic activity towards lipid substrates in the raw rice [38].

Three species of alkanes were detected in the FCRB, which together comprised the second most prevalent class of volatiles, accounting for 28.38% of the total volatile contents. Among them, tridecane, which is responsible for the grass odor in fermented black tea [39], was found in the highest concentration in the FCRB. This result was in agreement with the results of a previous study that found that tridecane was produced by chestnut trees during the full bloom stage [40]. However, alcohols comprised the second most abundant class of volatiles in the TFRB (21.60% of total), with 2-octanol representing the main alcohol component, similar to the alcohol composition in the FCRB.

In total, 22 species of benzenes were detected in the FCRB, accounting for the third largest proportion (15.88%) of volatiles. Pentylbenzene, an alkylbenzene identified in roast beef that has been described as having ethereal licorice notes [41], was the main benzene compound, while hexyl-benzene, which presents a slightly fruity aroma, was the second most abundant. However, only one species of benzene, i.e., toluene, was found in the TFRB. The third largest group of volatiles in the TFRB was aldehydes, including nine species that together comprised 17.96% of the total volatiles. Among them, nonanal, which confers a sweet citrus flavor, and benzaldehyde, a common aromatic aldehyde that imparts bitter almond, cherry, and nut odors [42], were the first- and second-highest concentration aldehydes in the TFRB, respectively.

The fourth largest class of volatiles in the FCRB were olefins, which included 18 compounds and accounted for 13.58% of total volatile contents. The three main olefins included 1-dodecene, (E)-2-octene, and 1-tridecene, all of which have been detected in anise-flavored spirits [43] and in irradiation-treated beef [44]. Apart from the above classes of volatiles, several esters such as 2-ethylhexyl acetate and ethyl benzoate, which impart a floral, fruity aroma [36], as well as aldehydes, organic acids, furans, and pyridines, were also identified in the FCRB. Ketones (7.63%) were the fourth most abundant volatile group, with 2-octanone, known for its botanical properties, the most abundant of these [45]. In addition to the above flavor-related volatiles, olefins were also detected in the TFRB, such as β-caryophyllene and santalene, the main compounds in *Cordia verbenacea* de Candolle, which is used as an anti-inflammatory and analgesic treatment for rheumatism [46]. Other studies have shown that the carbohydrate, amino acid, and lipid contents in the raw materials, in conjunction with the fermentation microbes and fermentation conditions, can together affect the formation of flavor-related metabolites and the final sensory profile of fermented beverages [36]. Since the fermentation starter microbes and fermentation conditions were identical between the FCRB and TFRB, the major differences in flavor-related volatiles can be reasonably attributed to the addition of chestnuts.

### 3.3. Antioxidant Activity of the Fermented Chestnut-Glutinous Rice Beverage (FCRB)

#### 3.3.1. Evaluation of FCRB Chemical Antioxidant Capacity In Vitro

Antioxidant activity can inhibit the production and accelerate the scavenging of reactive oxygen species (ROS) and free radicals to inhibit their oxidative damage of cellular macromolecules. Antioxidant capacity is commonly estimated by quantifying free radical scavenging activity, hydrogen production, and metal ion reducing capacity. Antioxidants are thus used to reduce the risk of cell damage and cell death related to ROS and free radical accumulation. Although there are many effective in vitro chemical methods for assessing antioxidant activity, each method also has shortcomings. Therefore, the use of multiple, complementary methods, including tests for DPPH^•^, hydroxyl radical (^•^OH), and ABTS^•^ radical scavenging, can together provide a more accurate picture of the antioxidant capacity and reducing power of the two fermented beverages.

**Figure 1:**
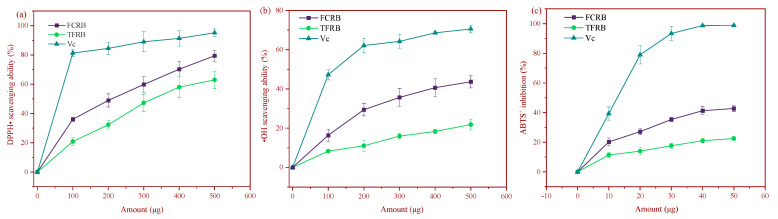
Chemical antioxidant capacity of FCRB or TFRB in vitro. (**a**) Scavenging ability of DPPH• by each fermented beverage; vitamin C (Vc) was used as the positive control. (**b**) Hydroxyl radicals scavenged by FCRB or TFRB. (**c**) ABTS^+^ radical scavenging activity of FCRB and TFRB. All data are means ± SD for three measurements.

Although exposure to FCRB or TFRB both resulted in DPPH^•^ free radical cleavage, FCRB displayed significantly higher activity than TFRB (Figure 1a), scavenging 79.4% and 62.5% of DPPH^•^, respectively, at 500 µg added amount (Figure 1a). The higher DPPH^•^ scavenging ability may be due to the combined effects of various organic acid compounds [47]. The wide diversity and similar properties of antioxidant chemical species makes it difficult to isolate and quantify individual antioxidants (i.e., parent compounds, glycosides, polymers, and various isomers) from a plant matrix or crude extract. Moreover, total antioxidant power is often more biologically meaningful for evaluating potential health benefits, since many antioxidants have cooperative activity [48]. Colorimetric Ferric ion reducing antioxidant power (FRAP) assays are a simple and reliable means of assessing “total reducing power” [48], in which reduction of ferric (Fe^3+^) ions to ferrous (Fe^2+^) ions at low pH induces ferrous-tripyridyltriazine complex formation (see Table S1 for standard curve). In agreement with the above antioxidant assays, FRAP assays indicated that FCRB had 448.12 ± 11.23 µmol/L total reducing power, which was significantly higher than that of TFRB (239.35 ± 15.11 µmol/L).

Hydroxyl radicals are among the most reactive ROS, and numerous methods for quantifying ^•^OH scavenging activity have been established. Among these methods, the Fenton reaction system, in particular, is a widely used and effective method to evaluate antioxidant properties of foods [49]. Fenton reaction assays indicated that both the FCRB and TFRB showed ^•^OH radical scavenging activity, but the FCRB, which used chestnuts for a third of its raw fermentation substrate, exhibited significantly greater scavenging than the TFRB did (Figure 1b). When 500 µg was the added amount, the FCRB scavenged approximately 2.0-fold more ^•^OH than the TFRB at the same concentration (Figure 1b). The ABTS assay is another commonly used method for screening the antioxidant ability of natural products and is applicable to both hydrophilic and lipophilic antioxidant systems [50]. Both the fermented beverages could scavenge ABTS^+^ radicals, but the FCRB showed significantly higher ABTS^+^ radical inhibition, although significantly less than that of ascorbic acid (vitamin C, Vc) (Figure 1c).

Taken together, these findings supported the likelihood that the addition of chestnuts in the FCRB resulted in higher in vitro chemical antioxidant capacity than that of the TFRB, which is consistent with the wide variety of vitamins and phenols that are reportedly enriched in chestnuts [31]. Although a significant decrease in the vitamin C content of chestnuts occurs during the steaming process, a proportion of the ascorbic acid is converted to dehydroascorbic acid, which retains some of its relatively potent antioxidant properties [24]. In addition, polyphenols bound to dietary fiber are released by the hydrolytic activity of microorganisms during fermentation [32]. Previous work in chestnut glutinous rice wine (banli mijiu, BLMJ) similarly identified three chestnut-specific phenols that potentially contributed to enhanced radical scavenging compared to that of traditional glutinous rice wine without chestnuts [11].

#### 3.3.2. Evaluation of the FCRB and TFRB Cellular Antioxidant Activity (CAA)

While the antioxidant potential of different substances can be determined in vitro, these above assays cannot be used to reliably predict antioxidant activity in biological contexts because of other physiological processes [51]. In order to account for cellular processes such as the uptake and metabolism in measuring antioxidant bioavailability, CAA assays were conducted to measure activity in live Caco-2 human colon adenocarcinoma cells.

##### Effects on Cell Viability under Oxidative Stress

**Figure 2:**
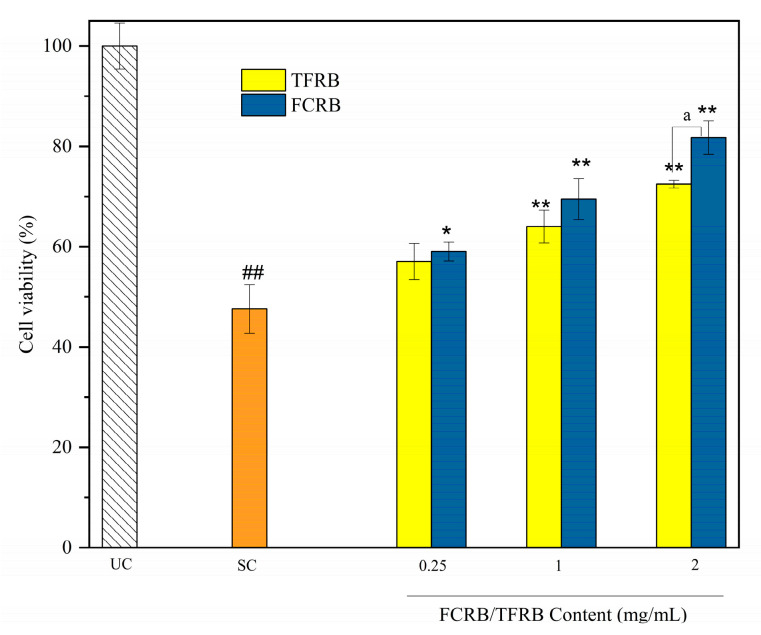
MTT assays of FCRB or TFRB effects on cytotoxicity in Caco-2 cells with H_2_O_2_-induced oxidative stress. UC, untreated controls; SC, stress controls treated with 1 mM H_2_O_2_ only. The results are presented as means ± SD (*n* = 3). ## *p* < 0.01 vs. UC; * *p* < 0.05, ** *p* < 0.01 vs. SC; ^a^ *p* < 0.01 vs. the TFRB (at the same concentration) as determined using the one-tailed *t* test.

In light of our in vitro antioxidant activity data, we next evaluated the bioaccessible fractions (BFs) of antioxidant compounds in the FCRB and TFRB in a Caco-2 cell model of the intestinal barrier. MTT tests were applied to determine the rate of mitochondrial metabolism as an indirect measure of cell viability and cytoprotection against oxidative stress of the BFs in the FCRB and TFRB. Caco-2 cells pretreated for 24 h with each respective beverage at a range of concentrations resulted in no obvious changes in cell viability compared to that of control cells, indicating that neither beverage significantly compromised cell integrity during the incubation period (Figure S1). However, after removing cells from each respective pretreatment and exposing them to H_2_O_2_ for 2 h, cell viability was significantly decreased to 47.62% that of the untreated controls (*p* < 0.01) (Figure 2). By contrast, cells pretreated with either fermented beverage at different concentrations (except 0.25 mg/mL TFRB) showed significantly higher cell viability under oxidative stress than stressed control cells without pretreatment. In addition, both fermented beverages led to an increasing percentage of viable cells with increasing fermented beverage concentration in the pretreatment. However, the bioavailable FCRB had stronger protective effects on mitochondrial enzyme activity than the TFRB at the same concentration, most obviously at 2 mg/mL (81.75% vs. 72.47%, *p* < 0.01) (Figure 2). Other studies have shown that polyphenols, flavonoids, and extracellular secretions of lactic acid bacteria can all protect Caco-2 cells against H_2_O_2_-induced oxidative stress [52,53]. We found that the FCRB contained different polyphenols and flavonoids than the TFRB, which could contribute to the observed differences in their cytoprotective effects.

##### Intracellular ROS Accumulation

**Figure 3:**
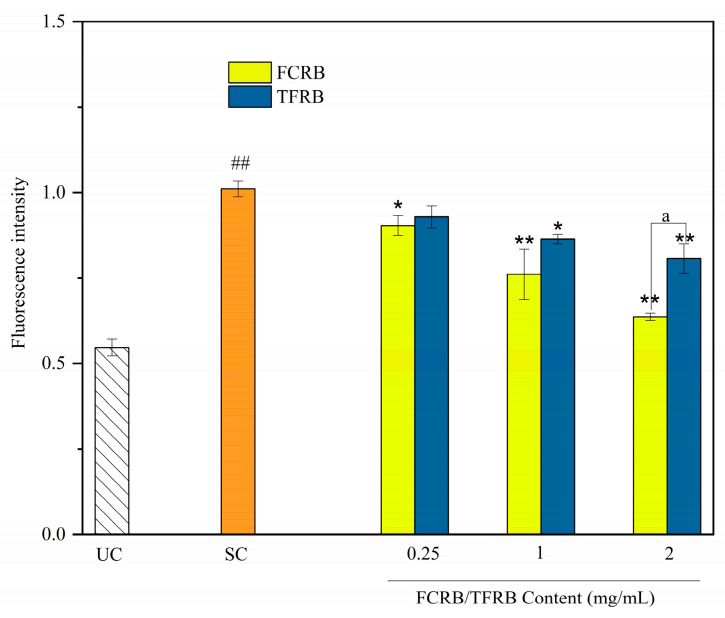
Effect of the FCRB or TFRB in H_2_O_2_-induced ROS generation in Caco-2 cells. The ROS levels were measured using the DCFH-DA probe. UC, untreated controls; SC, stress controls treated with 1 mM H_2_O_2_ only. The results are presented as means ± SD (*n* = 3). ## *p* < 0.01 vs. UC; * *p* < 0.05, ** *p* < 0.01 vs. SC; ^a^ *p* < 0.01 vs. TFRB (at the same concentration) as determined by using the one-tailed *t* test.

In addition to their essential functions in facilitating maintenance of redox homeostasis in cells, reactive oxygen species (ROS) can also function as secondary messenger signal molecules involved in cell growth and apoptosis processes [54]. Moreover, dysregulation or excessive ROS are well-known to contribute to the pathogenesis and development of numerous diseases [55]. Therefore, evaluating overall oxidative stress in cells by determining intracellular ROS levels can be informative of the cellular redox state. The measurement of intracellular ROS accumulation in cell cultures preincubated with the FCRB or TFRB then subjected to oxidative stress (Figure 3) showed that ROS accumulation significantly increased by approximately two-fold in cells challenged with H_2_O_2_ (1.011 ± 0.023) compared to control cells (0.547 ± 0.025) (*p* < 0.01). However, cells pretreated with either fermented beverage and subsequently exposed to oxidative stress had lower ROS levels (*p* < 0.05) than stressed cells with no pretreatment at FCRB concentrations as low as 0.25 mg/mL, but still had higher levels than that in cultures without oxidative stress, indicating that antioxidant metabolites in the bioavailable fraction of FCRB appeared sufficient to quench a significant proportion of excess ROS in cells.

Kang and colleagues showed that that some lactic acid bacteria, such as *Lactobacillus plantarum*, can activate both enzymatic and non-enzymatic defense systems in yeast cells, resulting in decreased intracellular ROS levels in yeast co-cultured with *L. plantarum* [56]. In addition, at 0.25 mg/mL and 1 mg/mL, FCRB and TFRB pretreatments resulted in similar ROS quenching, with the FCRB groups showing slightly lower trends of ROS accumulation. However, at 2 mg/mL, the ROS levels in the FCRB (0.637 ± 0.011) were significantly lower than that in the TFRB (0.808 ± 0.043, *p* < 0.01), further supporting that antioxidant metabolites were produced from chestnuts during fermentation but were absent in the TFRB. Cilla and coworkers identified flavonoids and monophenolics in sweet oranges that could attenuate ROS production induced by 2 h exposure to 200 µM H_2_O_2_ [52]. Our previous research similarly identified three monophenolic species produced during chestnut-rice wine fermentation that could significantly increase radical scavenging activity towards ABTS^+•^, •OH, and DPPH• [11]. In the current work, total phenolics and total flavonoids were significantly higher in the FCRB than in the TFRB, further supporting that the higher ROS quenching ability was due to the addition of chestnuts as a fermentation substrate.

##### Effects of FCRB on the Intracellular Antioxidant Enzyme Activity and Glutathione Levels

The total antioxidant capacity (T-AOC) is an index that describes the collective antioxidant activity of both proteins and small molecules. Measuring the effects of superoxide dismutase (SOD) and catalase (CAT), the first lines of enzymatic defense against oxidation [57], can show the contribution of each respective pathway for reducing ROS levels. In addition, glutathione (GSH) is among the most important antioxidants in cells due to its direct neutralization of peroxides and free radicals, and also contributes to maintaining pools of reduced ascorbate and α-tocopherol [58]. We thus compared intracellular T-AOC, SOD, CAT activity and GSH content between Caco-2 cell cultures treated or not with H_2_O_2_, and then also performed these assays in cells pretreated with FCRB or TFRB to identify the difference between the two fermented beverages.

**Figure 4:**
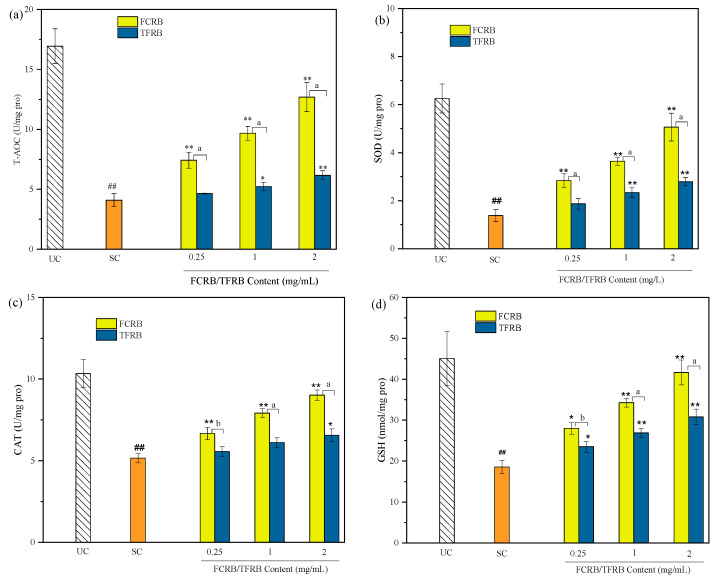
Effects of the FCRB or TFRB on total antioxidant capacity (T-AOC) (**a**), superoxide dismutase (SOD) (**b**), catalase (CAT) (**c**) and glutathione (GSH) levels (**d**) in Caco-2 cells. UC, untreated controls; SC, stress controls treated with 1 mM H_2_O_2_ only. The results are presented as means ± SD (*n* = 3). ## *p* < 0.01 vs. UC; * *p* < 0.05, ** *p* < 0.01 vs. SC; ^a^ *p* < 0.01 vs. TFRB (at the same concentration); ^b^ *p* < 0.05 vs. TFRB (at the same concentration) as determined by using the one-tailed *T* test.

The results showed that all of these indicators of antioxidant activity were significantly decreased in stressed cells (the SC group) compared to that in unstressed cells (the UC group) (*p* < 0.01). Specifically, T-AOC was 16.94 ± 1.45 U/mg protein in the UC group, and decreased to 4.09 ± 0.55 U/mg protein under hydrogen peroxide treatment in the SC group (Figure 4a). Similarly, SOD activity was 6.25 ± 0.61 U/mg protein in the UC group and only 1.38 ± 0.25 U/mg protein in the SC group (Figure 4b). However, 24 h pretreatment of Caco-2 cells with chestnut glutinous fermented beverage (FCRB) resulted in a significant increase in T-AOC, SOD, and CAT activity (*p* < 0.01) and GSH content (*p* < 0.05) upon treatment with H_2_O_2_ (Figure 4c,d). Notably, this increase followed a dose dependent trend, indicating that pretreatment with the FCRB could enhance antioxidant function in Caco-2 cells during oxidative stress (Figure 4). In addition, CAT and SOD activity and GSH content in the high-dose group (2 mg/mL) were close to the levels observed in untreated, non-stress controls (*p* < 0.01). For example, in Caco-2 cells pretreated with 2 mg/mL FCRB, the GSH content was 92.43% that in untreated cells (41.62 vs. 45.03 mM/mg protein). Pretreatment with the TFRB also resulted in higher T-AOC, SOD, and CAT activity and GSH content, although at significantly lower levels than that conferred by an equivalent concentration of FCRB (*p* < 0.01). For instance, pretreatment with 2 mg/mL FCRB resulted in 2.06-fold higher T-AOC activity than pretreatment with TFRB (12.69 vs. 6.16 U/mg protein).

These results supported that the FCRB could induce a higher antioxidant response in Caco-2 cells. These effects could be at least partially due to the nutrient composition of chestnuts. Since SOD and CAT are both metalloenzymes, they require iron, zinc, and/or manganese as co-factors for their function [57], and thus, the addition of chestnuts as a fermentation substrate could potentially increase the mineral content, especially iron and manganese, which have been reported as important micronutrients in chestnuts [59]. In addition, numerous phenolics and polysaccharides have been shown to confer antioxidant activity, potentially promoting human health [60], while acidic amino acids such as aspartate and glutamate can serve as proton donors for unpaired electrons, neutralizing free radicals [61]. The nutritional profiles shown here (Table 1) indicate that these compounds are abundant in chestnuts and may be retained in the final product after the fermentation process. In addition, probiotic LAB strains may be also partially responsible for antioxidant metabolite accumulation in the fermented beverages, since they harbor powerful redox systems and oxidative damage repair systems with abundant antioxidative enzymes [62].

##### The Effects of the FCRB on Apoptosis and Necrosis during Oxidative Stress in Caco-2 Cells

**Figure 5:**
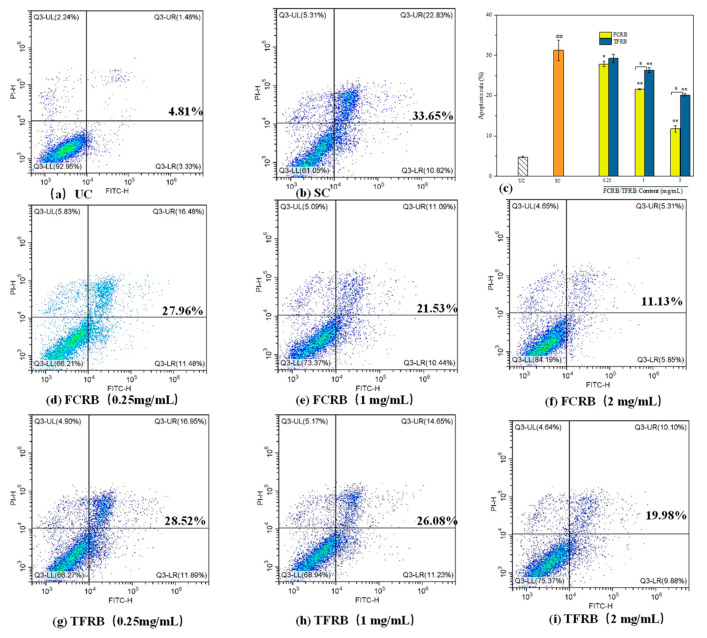
Effects of the FCRB on apoptosis and necrosis rates of Caco-2 cells under oxidative stress. (**a**) the apoptosis and necrosis rates of Caco-2 cells in non-stressed untreated controls (UC); (**b**) the apoptosis and necrosis rates of Caco-2 cells with 1 mM H_2_O_2_-only treatment group; (**c**) the apoptosis rate of Caco-2 cells treated with different concentration fermented beverage before 1 mM H_2_O_2_-treated. (**d**) the apoptosis and necrosis rate of Caco-2 cells treated with 0.25 mg/mL FCRB before 1 mM H_2_O_2_-treated; (**e**) the apoptosis and necrosis rate of Caco-2 cells treated with 1 mg/mL FCRB before 1 mM H_2_O_2_-treated; (**f**) the apoptosis and necrosis rate of Caco-2 cells treated with 2 mg/mL FCRB before 1 mM H_2_O_2_-treated; (**g**) the apoptosis and necrosis rate of Caco-2 cells treated with 0.25 mg/mL TFRB before 1 mM H_2_O_2_-treated; (**h**) the apoptosis and necrosis rate of Caco-2 cells treated with 1 mg/mL TFRB before 1 mM H_2_O_2_-treated; (**i**) the apoptosis and necrosis rate of Caco-2 cells treated with 2 mg/mL TFRB before 1 mM H_2_O_2_-treated. Apoptosis in Caco-2 cells was assessed via flow cytometry analysis Annexin V and PI staining. The results are presented as mean ± SD (*n* = 3). UC, untreated controls; SC, stress controls treated with 1 mM H_2_O_2_ only. ## *p* < 0.01 vs. UC; * *p* < 0.05, ** *p* < 0.01 vs. SC; ^a^ *p* < 0.01 vs. TFRB (at the same concentration) as determined by using the one-tailed T test (Q3-UL: necrotic cells; Q3-UR: late apoptotic cells; Q3-LL: normal cells; Q3-LR: early apoptotic cells).

Based on our results showing enhanced antioxidant activity in cells, we then investigated whether pretreatment with the FCRB also affected the induction of apoptosis or necrosis under oxidative stress using flow cytometry to quantify Annexin V/PI staining (Figure 5). The results indicated that 10.82% of cells entered early apoptosis after 2 h of exposure to H_2_O_2_ alone, while 22.83% entered late apoptosis, and 5.31% underwent necrosis, which were obviously higher levels than those in non-stressed untreated controls (Figure 5a,b). Similarly, the proportion of viable cells was 61.05% in the H_2_O_2_-only treatment group, 31.9% lower than that in the UC group (Figure 5a,b). However, 24 h exposure to FCRB prior to stress induction led to significantly lower levels of apoptotic and necrotic cells (Figure 5c). At 0.25 mg/mL FCRB, 66.21% of cells remained viable under peroxide treatment, with 11.48% and 16.48% exhibiting early or late apoptosis, respectively (Figure 5d). At 1 mg/mL FCRB pretreatment, 73.37% were viable, with even lower proportions of early (10.44%) and late (11.09%) apoptosis (Figure 5e). However, following incubation with 2 mg/mL FCRB, the percentage of viable cells reached 84.19% under H_2_O_2_, close to that in the non-stressed UC group (Figure 5c,f), suggesting that FCRB could promote the inhibition of H_2_O_2_-induced apoptosis in Caco-2 cells in a dose-dependent manner.

Notably, the necrosis rates were 5.83%, 5.09%, and 4.65% in the low-, medium-, and high-FCRB treatment groups (Figure 5d–f), respectively, thus showing an apparent decreasing trend, though not significantly different from that in stressed cells with no exposure to the FCRB (Figure 5b). Although TFRB pretreatment also resulted in decreased levels of apoptotic and necrotic cells, the effects were substantially less pronounced than that conferred by the FCRB at 1 mg/mL and 2 mg/mL concentrations (i.e., apoptosis rates of 19.98% vs. 11.13% and 75.37% vs. 84.19% viable cells at 2 mg/mL TFRB vs. FCRB, *p* < 0.01) (Figure 5c,g–i).

Previous studies have shown that some lactic acid bacteria, such as *L. paracasei* and *P. pentosaceus,* are mainly protected against oxidative toxicity by O_2_-consuming enzymes and redox and repair systems [62]. The enhanced resistance to apoptosis we observed in Caco-2 cells following pretreatment with the FCRB may also be a result of chestnut addition, either through the retention of phenolics and polysaccharides from the raw substrate or through the accumulation of metabolites produced during fermentation, such as dehydroascorbic acid or monophenols derived from soluble fiber-bound polyphenols, which aligns well with findings in other studies [24,32,63].

## 4. Conclusions

In this paper, the metabolite composition and antioxidant or cell-protective properties of the TFRB and FCRB were characterized in vitro to determine the effects of chestnuts on LAB fermentation of glutinous rice. Generally, the FCRB has higher organic acid but lower amino acid contents than the TFRB (Table 1). Although chestnuts comprised only one quarter of the raw fermentation substrate, both the species and quantities of volatile organic compounds (VOCs) significantly differed between the FCRB and TFRB (Table 2). In addition to these changes in VOC and nutrient contents, the addition of chestnuts also led to enhanced scavenging activity towards DPPH•, •OH, and ABTS^+•^ radicals, and increased the total reducing power of the FCRB over that of the TFRB (Figure 1). Cell viability tests showed that neither fermented beverage induced cytotoxic effects in Caco-2 cells (Figure S1). Subsequent MTT assays indicated that pretreatment with 2 mg/mL FCRB provides significantly greater protection against peroxide-induced cytotoxicity than an equivalent pretreatment with the TFRB in Caco-2 cells (Figure 2). Although ROS levels increased by almost 2-fold following treatment with hydrogen peroxide, ROS can be largely restored to levels comparable with untreated controls by the addition of the FCRB to the Caco-2 cell cultures (Figure 3). Furthermore, pre-incubation with either fermented beverage can promote the activity of antioxidant enzymes, SOD and CAT (and T-AOC), and lead to elevated GSH contents, with the FCRB pretreatment resulting in significantly greater effects than the TFRB at moderate and high doses (Figure 4). In addition, the FCRB pretreatment also results in greater resistance to apoptosis than the TFRB in Caco-2 cells (Figure 5). These cumulative findings show that supplementing the TFRB with chestnuts in the fermentation leads to increased production of VOCs and amino acids, and enhances antioxidant activity in vitro and in cells.

## Figures and Tables

**Table 1:** The amino acids species and contents in the FCRB and TFRB.

Names	FCRB (mg/mL)	TFRB (mg/mL)	Names	FCRB (mg/L)	TFRB (smg/L)
Asp	276.543 ± 11.352 ^a^	201.364 ± 9.852 ^b^	Leu	125.211 ± 6.112 ^a^	210.366 ± 5.261 ^b^
Ser	91.245 ± 3.745 ^a^	185.343 ± 2.011 ^b^	Thr	71.322 ± 3.214 ^a^	130.651 ± 9.889 ^b^
Glu	375.514 ± 16.784 ^a^	409.322 ± 25.321 ^a^	Phe	106.255 ± 12.333 ^a^	265.112 ± 5.332 ^b^
Gly	87.417 ± 6.697 ^a^	233.364 ± 13.452 ^b^	Lys	81.233 ± 4.356 ^a^	206.355 ± 12.114 ^b^
Ala	121.353 ± 4.003 ^a^	265.411 ± 12.342 ^b^	Val	143.112 ± 20.112 ^a^	251.462 ± 6.004 ^b^
Cys	42.611 ± 2.988 ^a^	50.115 ± 4.350 ^a^	Met	52.233 ± 2.331 ^a^	112.334 ± 12.112 ^b^
Tyr	54.213 ± 2.744 ^a^	251.355 ± 12.258 ^b^	Umami FAAs	652.057 ± 31.224 ^a^	610.686 ± 42.366 ^a^
His	223.911 ± 33.441 ^a^	226.009 ± 15.331 ^a^	Bitter FAAs	995.304 ± 19.356 ^a^	1932.607 ± 88.356 ^b^
Pro	102.233 ± 4.554 ^a^	266.400 ± 2.134 ^b^	Sweet FAAs	525.803 ± 22.331 ^a^	1193.503 ± 90.522 ^b^
Arg	184.256 ± 8.311 ^a^	346.351 ± 20.005 ^b^	EAAs	880.390 ± 25.336 ^a^	1577.886 ± 73.443 ^b^
Ile	77.113 ± 3.335 ^a^	175.597 ± 3.110 ^b^	SUM FAAs	2215.775 ± 98.455 ^a^	3786.911 ± 162.205 ^b^

All values are means of triplicate determinations ± SD. Means with different letters are significantly (*p* < 0.05) different from other values in the same amino acid row. Umami FAAs (Glu and Asp), sweet FAAs (Thr, Ser, Gly, Ala, Pro, and Met), and bitter FAAs (Tyr, Ile, Leu, Val, Phe, Lys, His, and Arg). EAAs: essential amino acids (His, Ile, Leu, Lys, Met, Phe, Thr and Val).

**Table 2:** Flavor materials in the FCRB and TFRB identified using headspace solid phase microextraction-gas chromatography mass spectrometry (HS-SPME/GC-MS).

Names	Retention Time (min)	Relative Content (ng/L)		Matched Degree
		FCRB	TFRB	
Alcohols		8	10	
Isoamyl alcohol	8.776	0.837	2.505	90
2-Octanol	24.040	12.512	10.275	90
Benzene ethanol	31.974	1.12	1.122	91
1-Propanol	5.4461	0.095	-	85
2-Methyl-1-propanol	6.268	0.111	-	83
1-Heptanol	21.491	0.113	-	91
1-Octanol	28.946	0.752	-	91
2-Nonanol	31.146	0.957	-	83
2-Methyl-1-butanol	11.116	-	0.966	90
1-Octen-3-ol	24.034	-	0.345	90
1-Decanol	36.757	-	0.431	95
2,4-Hexadien-1-ol	45.609	-	0.315	93
2-Nonen-1-ol	50.318	-	0.502	92
Cyclooctanemethanol	51.943	-	0.157	88
trans-3-Methylcyclohexanol	57.494	-	0.192	92
Total content		16.497	16.810	
Esters		11	3	
Ethyl hexadecanoate	67.47	0.96	24.799	90
Ethyl tetradecanoate	62.635	0.005	4.492	98
Ethyl Acetate	6.062	0.042	-	90
Ethyl 4-hydroxy-dl-mandelate	30.646	0.015	-	78
2-Ethylhexyl acetate	33.526	0.218	-	60
1-Methylheptyl acetate	33.581	0.106	-	75
Ethyl benzoate	36.203	0.289	-	94
Ethyl caprate	49.559	0.044	-	93
Dodecyl-hexanoate	53.551	0.026	-	65
Butyl butyryl lactate	56.897	0.173	-	80
Ethyl palmitate	67.469	0.965	-	98
Methyl linolate	71.176	-	3.077	80
Total content		2.843	32.368	
Aldehyde		5	9	
2-Heptenal	20.55	0.067	1.511	97
Benzaldehyde	21.00	0.316	3.460	94
(E)-2-Octenal	28.049	0.065	1.118	84
Furfural	12.903	0.779	-	90
p-Tolualdehyde	41.147	0.036	-	75
Hexanal	13.901	-	1.396	80
Nonanal	28.593	-	4.398	85
(E)-2-Nonenal	32.467	-	0.611	90
α-Ethyl-benzeneacetaldehyde	42.375	-	0.241	92
Tetradecanal	65.503	-	0.985	94
cis-11-Hexadecenal	70.252	-	0.222	97
Total content		1.263	13.942	
Benzenes		22	1	
Toluene	10.05	0.161	0.242	95
Ethylbenzene	14.551	0.035	-	87
1,3-Dimethyl-benzene	15.050	0.042	-	94
1,2-Dimethyl-benzene	16.242	0.043	-	95
Propyl-benzene	20.396	0.051	-	90
1-Methoxypropylbenzene	28.492	0.103	-	85
1-Methyl-3-(1-methylethyl)-benzene	32.213	0.111	-	83
1,2,3,4-Tetramethyl-benzene	32.483	0.121	-	95
1-Isopropyl-2-methyl-benzene	34.051	0.279	-	85
2,5-Dimethoxyphenylethene	34.517	0.367	-	90
Pentyl- benzene	35.179	2.712	-	93
1-Methyl-4-(1-methylpropyl)- benzene	35.313	0.304	-	85
1-Methyl-4-butyl benzene	35.809	0.763	-	88
1-Methyl-2-(1-ethylpropyl)- benzene	40.171	0.693	-	94
1,4-Dimethyl-2-(2-methylpropyl)- Benzene	41.397	0.194	-	93
1,3,5-Triethyl- benzene	41.536	0.032	-	88
(1-Ethylbutyl)-benzene	42.085	0.026	-	85
Hexyl-benzene	42.72	1.377	-	90
(1,3-Dimethylbutyl)- benzene	43.035	0.828	-	86
1-Ethyl-4-(2-methylpropyl)- benzene	43.348	0.08	-	95
2-Ethyl-P-Xylene	47.016	0.052	-	86
Heptyl-benzene	48.277	0.157	-	83
Total content		8.531	0.242	
Olefins		18	5	
1–Octene	10.95	0.530	-	96
(E)-2-Octene	11.616	1.175	-	96
2-Methyl-1-Octene	15.891	0.024	-	81
D-Limonene	26.006	0.123	-	98
5-Undecene	31.326	0.332	-	95
(E) -1-Phenyl-1-butene	32.852	0.049	-	95
2,5-Dimethylstyrene	34.571	0.367	-	85
1-Dodecene	37.91	1.442	-	95
(E) -3-Dodecene	38.211	0.262	-	95
(Z)-2- Dodecene	38.333	0.072	-	97
Trans-1-phenyl-1-pentene	40.391	0.073	-	90
(E)-6-Tridecene	44.211	0.234	-	96
(E)-5-Tridecene	44.486	0.269	-	97
1-Tridecene	44.587	1.232	-	97
(E)-3-Tridecene	44.889	0.098	-	87
(Z)-2-Tridecene	45.289	0.624	-	92
(Z)-3-Tridecene	45.791	0.352	-	95
4-Propylindene	46.056	0.037	-	89
1,3-cis, 5-cis-Octatriene	24.529	-	0.165	85
2,6,10-Trimethyl-dodecane	29.279	-	0.232	90
β-Caryophyllene	49.742	-	2.148	95
D-Cadinene	55.993	-	0.618	97
1,7-Dimethyl-7-(4-methyl-3-pentenyl)-tricyclo [2.2.1.0(2,6)] heptane	60.235	-	1.625	92
Total content		7.295	4.788	
Ketones		3	3	
6-Methyl-2-heptanone	23.266	0.018	-	77
1-Phenylethanone	28.663	0.148	-	93
2-Nonanone	30.322	0.697	-	97
3,6-dimethyloxan-2-one	14.688	-	0.249	95
2-Octanone	21.663	-	5.364	97
3,7-Dihydro-1,3,7-trimethyl-1H-Purine-2,6-dione	65.081	-	0.324	95
Total content		0.863	5.937	
Alkanes		3	5	
Dodecane	38.594	1.008	-	96
Tridecane	45.098	14.187	-	98
Tetradecane	49.873	0.048	-	94
2,4-Dimethyl-heptane,	11.668	-	0.172	90
3,3-Dimethyl-hexane	26.109	-	0.202	92
2,3,5-Trimethyl-decane	36.420	-	0.291	94
Undecane	49.935	-	0.767	95
10-Methylnonadecane	54.347	-	0.254	94
Total content		15.243	1.686	
Organic acids		1	1	
Benzoic acid	35.059	0.179	-	86
Undecanoic acid	67.879	-	1.786	90
Total content		0.179	1.786	
Others		3	1	
2-Methylbenzofuran	31.669	0.211	-	96
2-Ethyl-5-methylpyridine	34.835	0.336	-	75
Naphthalene	37.552	0.446	-	95
Phthalan	37.341	-	0.268	90
Total content		0.993	0.268	

”-”not detected.

## Data Availability

Data are contained within the article or Supplementary Material.

## Supplementary Materials

The following supporting information can be downloaded at: https://www.mdpi.com/article/10.3390/foods12010164/s1, Figure S1: MTT assays of FCRB or TFRB cytotoxicity towards Caco-2 cells. The results are presented as means ± SD (*n* = 3). Viability of Caco-2 cells incubated for 24 h with different concentration of the FCRB (a) or the TFRB (b), Table S1: Linear regression of gallic acid, rutin, and ferrous sulfate.

## References

[1] Villaño D., Gironés-Vilapana A., García-Viguera C., Moreno D.A., Galanakis C.M. (2022). Development of functional foods. Innovation Strategies in the Food Industry.

[2] Bogue J., Collins O., Troy A.J., Bagchi D., Nair S. (2017). Market analysis and concept development of functional foods. Developing New Functional Food and Nutraceutical Products.

[3] Masoumi S.J., Mehrabani D., Saberifiroozi M., Fattahi M.R., Moradi F., Najafi M. (2021). The effect of yogurt fortified with *Lactobacillus acidophilus* and *Bifidobacterium* sp. probiotic in patients with lactose intolerance. Food Sci. Nutr..

[4] Ziarno M., Zaręba D., Henn E., Margas E., Nowak M. (2019). Properties of non-dairy gluten-free millet-based fermented beverages developed with yoghurt cultures. J. Food Nutr. Res..

[5] Corbo M.R., Bevilacqua A., Petruzzi L., Casanova F.P., Sinigaglia M. (2015). Functional beverages: The emerging side of functional foods. Compr. Rev. Food Sci. Food Saf..

[6] Waters D.M., Mauch A., Coffey A., Arendt E.K., Zannini E. (2015). Lactic acid bacteria as a cell factory for the delivery of functional biomolecules and ingredients in cereal-based beverages: A review. Crit. Rev. Food Sci. Nutr..

[7] Yang F., Huang X., Zhang C., Zhang M., Huang C., Yang H. (2018). Amino acid composition and nutritional value evaluation of Chinese chestnut (*Castanea mollissima* Blume) and its protein subunit. RSC Adv..

[8] Murthy H.N., Bapat V.A., Murthy H., Bapat V. (2020). Importance of underutilized fruits and nuts. Bioactive Compounds in Underutilized Fruits and Nuts.

[9] Vasconcelos M.C.D., Eduardo AS Rosa R.B., Ferreira-Cardoso J.V. (2010). Composition of european chestnut (*Castanea sativa* Mill.) and association with health effects: Fresh and processed products. J. Sci. Food Agric..

[10] Murado M.A., Pastrana L., Vázquez J.A., Mirón J., González M.P. (2008). Alcoholic chestnut fermentation in mixed culture. Compatibility criteria between *Aspergillus oryzae* and *Saccharomyces cerevisiae* strains. Bioresour. Technol..

[11] Zou J., Ge Y., Zhang Y., Ding M., Li K., Lin Y., Chang X., Cao F., Qian Y. (2022). Changes in flavor-and aroma-related fermentation metabolites and antioxidant activity of glutinous rice wine supplemented with Chinese chestnut (*Castanea mollissima* Blume). Fermentation.

[12] Gaya P., Peirotén Á., Landete J.M. (2017). Transformation of plant isoflavones into bioactive isoflavones by lactic acid bacteria and bifidobacteria. J. Funct. Foods.

[13] Escrivá L., Manyes L., Vila-Donat P., Font G., Meca G., Lozano M. (2021). Bioaccessibility and bioavailability of bioactive compounds from yellow mustard flour and milk whey fermented with lactic acid bacteria. Food Funct..

[14] Arena M.P., Capozzi V., Russo P., Drider D., Spano G., Fiocco D. (2018). Immunobiosis and probiosis: Antimicrobial activity of lactic acid bacteria with a focus on their antiviral and antifungal properties. Appl. Microbiol. Biotechnol..

[15] Korcz E., Kerényi Z., Varga L. (2018). Dietary fibers, prebiotics, and exopolysaccharides produced by lactic acid bacteria: Potential health benefits with special regard to cholesterol-lowering effects. Food Funct..

[16] Sankar G.S., Sankar S.S., Subrata S., Venkatachalam S., Chang P.S. (2018). Use of a potential probiotic, *Lactobacillus plantarum* L7, for the preparation of a rice-based fermented beverage. Front. Microbiol..

[17] Yi R.K., Peng P., Zhang J., Du M.Y., Lan L.X., Qian Y., Zhou J., Zhao X. (2019). *Lactobacillus plantarum* CQPC02-fermented soybean milk improves loperamide-induced constipation in mice. J. Med. Food.

[18] Angelescu I.R., Zamfir M., Stancu M.M., Grosu-Tudor S.S. (2019). Identification and probiotic properties of *lactobacilli* isolated from two different fermented beverages. Ann. Microbiol..

[19] Salmerón I. (2017). Fermented cereal beverages: From probiotic, prebiotic and synbiotic towards Nanoscience designed healthy drinks. Lett. Appl. Microbiol..

[20] Peyer L.C., Zannini E., Arendt E.K. (2016). Lactic acid bacteria as sensory biomodulators for fermented cereal-based beverages. Trends Food Sci. Technol..

[21] Hu Y., Chen X., Chang X., Wang Y., Zou J. (2022). Screening of lactic acid bacteria from jiuqu and its application in the fermentation of chestnut glutinous rice beverage. Sci. Technol. Food Ind..

[22] Lingua M.S., Fabani M.P., Wunderlin D.A., Baroni M.V. (2016). In vivo antioxidant activity of grape, pomace and wine from three red varieties grown in Argentina: Its relationship to phenolic profile. J. Funct. Foods.

[23] Chen H., Xiao G., Xu Y., Yu Y., Wu J., Zou B. (2019). High hydrostatic pressure and co-fermentation by *Lactobacillus rhamnosus* and *Gluconacetobacter xylinus* improve flavor of yacon-litchi-longan juice. Foods.

[24] Barros A.I., Nunes F.M., Gonçalves B., Bennett R.N., Silva A.P. (2011). Effect of cooking on total vitamin C contents and antioxidant activity of sweet chestnuts (*Castanea sativa* Mill.). Food Chem..

[25] Ye C.-L., Hu W.-L., Dai D.-H. (2011). Extraction of polysaccharides and the antioxidant activity from the seeds of *Plantago asiatica* L.. Int. J. Biol. Macromol..

[26] Marsh A.J., Hill C., Ross R.P., Cotter P.D. (2014). Fermented beverages with health-promoting potential: Past and future perspectives. Trends Food Sci. Technol..

[27] Ignat M.V., Salanta L.C., Pop O.L., Pop C.R., Tofana M., Mudura E., Coldea T.E., Borsa A., Pasqualone A. (2020). Current functionality and potential improvements of non-alcoholic fermented cereal beverages. Foods.

[28] O’Bryan C., Crandall P., Ricke S., Ndahetuye J., Taylor M.T. (2015). Lactic acid bacteria (LAB) as antimicrobials in food products: Types and mechanisms of action. Handbook of Natural Antimicrobials for Food Safety and Quality.

[29] Coban H.B. (2020). Organic acids as antimicrobial food agents: Applications and microbial productions. Bioproc. Biosyst. Eng..

[30] Gonçalves B., Borges O., Costa H.S., Bennett R., Santos M., Silva A.P. (2010). Metabolite composition of chestnut (*Castanea sativa* Mill.) upon cooking: Proximate analysis, fibre, organic acids and phenolics. Food Chem..

[31] Vekiari S., Gordon M., García-Macías P., Labrinea H. (2008). Extraction and determination of ellagic acid contentin chestnut bark and fruit. Food Chem..

[32] Xie J., Liu S., Dong R., Xie J., Chen Y., Peng G., Liao W., Xue P., Feng L., Yu Q. (2021). Bound polyphenols from insoluble dietary fiber of defatted rice bran by solid-state fermentation with trichoderma viride: Profile, activity, and release mechanism. J. Agric. Food Chem..

[33] Han N.D., Cheng J., Delannoy-Bruno O., Webber D., Terrapon N., Henrissat B., Rodionov D.A., Arzamasov A.A., Osterman A.L., Hayashi D.K. (2022). Microbial liberation of N-methylserotonin from orange fiber in gnotobiotic mice and humans. Cell.

[34] Li-Chan E.C., Cheung I.W., Mine Y., Li-Chan E., Jiang B. (2010). Flavor-active properties of amino acids, peptides, and proteins. Bioactive Proteins and Peptides as Functional Foods and Nutraceuticals.

[35] Liu S., Yang L., Zhou Y., He S., Li J., Sun H., Yao S., Xu S. (2019). Effect of mixed moulds starters on volatile flavor compounds in rice wine. LWT.

[36] Yang Y., Xia Y., Wang G., Yu J., Ai L. (2017). Effect of mixed yeast starter on volatile flavor compounds in Chinese rice wine during different brewing stages. LWT.

[37] Buttery R.G., Stern D.J., Ling L.C. (1994). Studies on flavor volatiles of some sweet corn products. J. Agric. Food Chem..

[38] Ding X., Wu C., Huang J., Zhou R. (2015). Changes in volatile compounds of Chinese Luzhou-flavor liquor during the fermentation and distillation process. J. Food Sci..

[39] Kim D.-S., Lee J.T., Hong S.J., Cho J.-J., Shin E.-C. (2019). Thermal coursed effect of comprehensive changes in the flavor/taste of *Cynanchi wilfordii*. J. Food Sci..

[40] Liang J., Zhao Y., Chang X. (2014). Changes of aroma components in chestnut flower at different flowering stage. J. Fruit Sci..

[41] Min D.B., Ina K., Peterson R., Chang S. (1977). The alkylbenzenes in roast beef. J. Food Sci..

[42] Chen S., Wang C., Qian M., Li Z., Xu Y. (2019). Characterization of the key aroma compounds in aged Chinese rice wine by comparative aroma extract dilution analysis, quantitative measurements, aroma recombination, and omission studies. J. Agric. Food Chem..

[43] Jurado J., Ballesteros O., Alcazar A., Pablos F., Martín M., Vilchez J., Navalon A. (2007). Characterization of aniseed-flavoured spirit drinks by headspace solid-phase microextraction gas chromatography–mass spectrometry and chemometrics. Talanta.

[44] Cha Y.-J., Kim H., Park S.-Y., Kim S.-J., You Y.-J. (2000). Identification of irradiation-induced volatile flavor compounds in beef. J. Korean Soc. Food Sci. Nutr..

[45] Xu X., Xu R., Jia Q., Feng T., Huang Q., Ho C.-T., Song S. (2019). Identification of dihydro-β-ionone as a key aroma compound in addition to C8 ketones and alcohols in *Volvariella volvacea* mushroom. Food Chem..

[46] Quispe-Condori S., Foglio M.A., Rosa P.T., Meireles M.A.A. (2008). Obtaining β-caryophyllene from *Cordia verbenacea* de Candolle by supercritical fluid extraction. J. Supercrit. Fluids.

[47] Aung T., Eun J.-B. (2021). Production and characterization of a novel beverage from laver (*Porphyra dentata*) through fermentation with kombucha consortium. Food Chem..

[48] Amamcharla J.K., Metzger L.E. (2014). Modification of the ferric reducing antioxidant power (FRAP) assay to determine the susceptibility of raw milk to oxidation. Int. Dairy J..

[49] Kremer M. (1999). Mechanism of the Fenton reaction. Evidence for a new intermediate. Phys. Chem. Chem. Phys..

[50] Kandi S., Charles A. (2019). In vitro antioxidant activity of Kyoho grape extracts in DPPH and ABTS assays: Estimation methods for EC50 using advanced statistical programs. Food Chem..

[51] Gulcin İ. (2020). Antioxidants and antioxidant methods: An updated overview. Arch. Toxicol..

[52] Cilla A., Rodrigo M.J., Zacarías L., De Ancos B., Sánchez-Moreno C., Barberá R., Alegría A. (2018). Protective effect of bioaccessible fractions of citrus fruit pulps against H_2_O_2_-induced oxidative stress in Caco-2 cells. Food Res. Int..

[53] Vasiee A., Falah F., Behbahani B.A., Tabatabaee-Yazdi F. (2020). Probiotic characterization of *Pediococcus* strains isolated from Iranian cereal-dairy fermented product: Interaction with pathogenic bacteria and the enteric cell line Caco-2. J. Biosci. Bioeng..

[54] Rhee S.G. (2006). H_2_O_2_, a necessary evil for cell signaling. Science.

[55] Wang L., Wise J.T., Zhang Z., Shi X. (2016). Progress and prospects of reactive oxygen species in metal carcinogenesis. Curr. Pharmacol. Rep..

[56] Kang X., Gao Z., Zheng L., Zhang X., Li H. (2021). Regulation of *Lactobacillus plantarum* on the reactive oxygen species related metabolisms of *Saccharomyces cerevisiae*. LWT.

[57] Ighodaro O., Akinloye O. (2018). First line defence antioxidants-superoxide dismutase (SOD), catalase (CAT) and glutathione peroxidase (GPX): Their fundamental role in the entire antioxidant defence grid. Alex. J. Med..

[58] Giustarini D., Tsikas D., Colombo G., Milzani A., Dalle-Donne I., Fanti P., Rossi R. (2016). Pitfalls in the analysis of the physiological antioxidant glutathione (GSH) and its disulfide (GSSG) in biological samples: An elephant in the room. J. Chromatogr. B.

[59] De Vasconcelos M.d.C.B., Nunes F., Viguera C.G., Bennett R.N., Rosa E.A., Ferreira-Cardoso J.V. (2010). Industrial processing effects on chestnut fruits (*Castanea sativa* Mill.) 3. Minerals, free sugars, carotenoids and antioxidant vitamins. Int. J. Food Sci. Technol..

[60] De Vasconcelos M.d.C.B., Richard B., Stéphane Q., Rémi J. (2010). Evaluating the potential of chestnut (*Castanea sativa* Mill.) fruit pericarp and integument as a source of tocopherols, pigments and polyphenols. Ind. Crops Prod..

[61] Feng Y.X., Ruan G.R., Jin F., Xu J., Wang F.J. (2018). Purification, identification, and synthesis of five novel antioxidant peptides from Chinese chestnut (*Castanea mollissima* Blume) protein hydrolysates. LWT.

[62] Feng T., Wang J. (2020). Oxidative stress tolerance and antioxidant capacity of lactic acid bacteria as probiotic: A systematic review. Gut Microbes.

[63] Hu M., Yang X., Chang X. (2021). Bioactive phenolic components and potential health effects of chestnut shell: A review. J. Food Biochem..

