# Supplementary material for: Chestnuts in Fermented Rice Beverages Increase Metabolite Diversity and Antioxidant Activity While Reducing Cellular Oxidative Damage
Source: Foods. 2022 Dec 28;12(1):164. doi: 10.3390/foods12010164 (PMC9818290; doi:10.3390/foods12010164)
Supplement: Supplementary file 1 [file foods-12-00164-s001.zip › Figure S1.pdf]

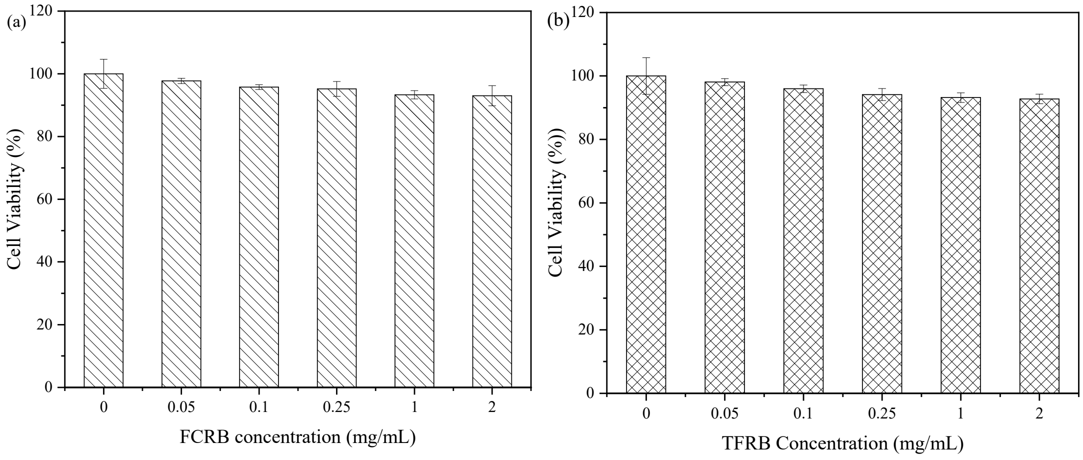

Figure S1. MTT assays of FCRB or TFRB cytotoxicity towards Caco-2 cells. The results are presented as means ± SD (n = 3). Viability of Caco-2 cells incubated for 24h with different concentration of FCRB (a) or TFRB (b).
