# Supplementary material for: Chestnuts in Fermented Rice Beverages Increase Metabolite Diversity and Antioxidant Activity While Reducing Cellular Oxidative Damage
Source: Foods. 2022 Dec 28;12(1):164. doi: 10.3390/foods12010164 (PMC9818290; doi:10.3390/foods12010164)
Supplement: Supplementary file 1 [file foods-12-00164-s001.zip › Table S1.pdf]

Table S1. Linear regression of gallic acid, rutin and ferrous sulfate

Name	Regression Equation	R^2
Gallic acid	Y=0.0057X-0.0067	0.9977
Rutin	Y=0.0133X-0.0042	0.9978
Ferrous sulfate	Y=0.997X+0.0163	0.9984
